# Supplementary material for: Targeting dCas9‐SunTag to a Susceptibility Gene Promoter Is Sufficient for CRISPR Interference
Source: Plant Direct. 2026 Feb 6;10(2):e70128. doi: 10.1002/pld3.70128 (PMC12877719; doi:10.1002/pld3.70128)
Supplement: Supplementary file 1 — Figure S1: nCBP‐1 promoter cytosine methylation landscape. Percent methylated cytosines across the nCBP‐1 promoter ampBS‐seq window for epigenome edited transgenic line #80 and the ΔgRNA control line #34. Each row within a genotype corresponds to an independent biological replicate. Figure S2: nCBP‐2 promoter cytosine methylation landscape. Percent methylated cytosines across the nCBP‐2 promoter ampBS‐seq window for epigenome edited transgenic line #80 and the ΔgRNA control line #34. Each row within a genotype corresponds to an independent biological replicate. Figure S3: MeSWEET10a promoter cytosine methylation landscape. Percent methylated cytosines across the MeSWEET10a promoter ampBS‐seq window for epigenome edited transgenic line #80 and the ΔgRNA control line #34. Each row within a genotype corresponds to an independent biological replicate. Figure S4: (a) Observed cycle quantitation (Cq) values for nCBP1 and nCBP2 across from across five qPCR experiments examining tissue culture leaf, greenhouse leaf, and CBSV‐infected storage root material. Threshold values for Cq selection were chosen based on those automatically selected by qPCR software for nCBP‐2 and then applied to nCBP‐1. (b) Differences in nCBP‐1 and nCBP‐2 Cq from each tissue sample used in (a). [file PLD3-10-e70128-s001.pdf]

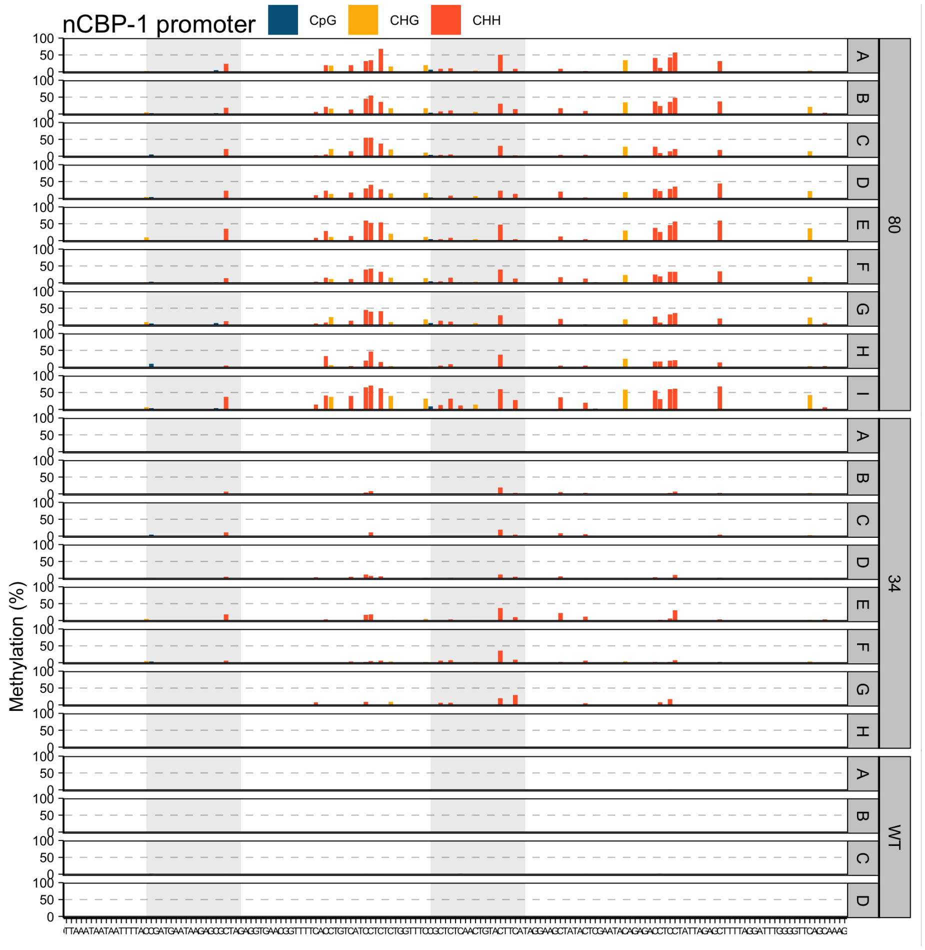

Figure S1. *nCBP-1* promoter cytosine methylation landscape

Percent methylated cytosines across the *nCBP-1* promoter ampBS-seq window for epigenome edited transgenic line #80 and the ∆gRNA control line #34. Each row within a genotype corresponds to an independent biological replicate.

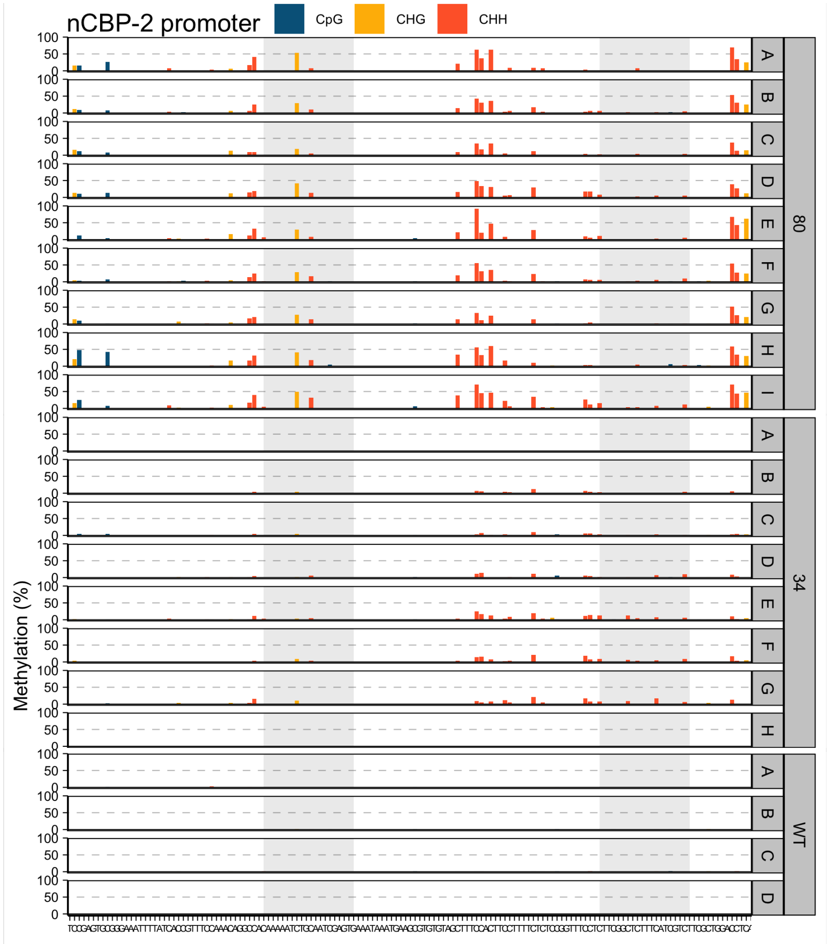

Figure S2. *nCBP-2* promoter cytosine methylation landscape

Percent methylated cytosines across the *nCBP-2* promoter ampBS-seq window for epigenome edited transgenic line #80 and the ∆gRNA control line #34. Each row within a genotype corresponds to an independent biological replicate.

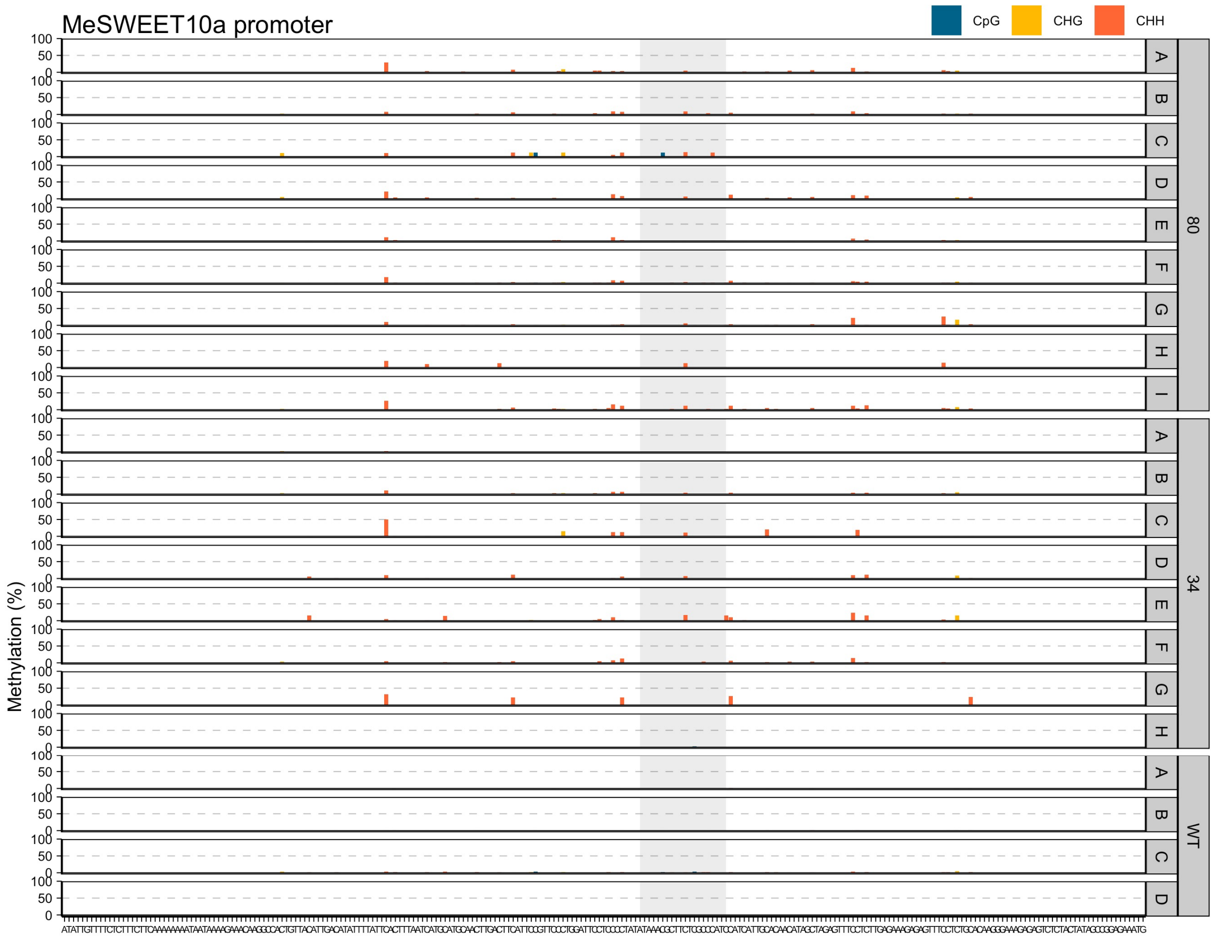

Figure S3. *MeSWEET10a* promoter cytosine methylation landscape

Percent methylated cytosines across the *MeSWEET10a* promoter ampBS-seq window for epigenome edited transgenic line #80 and the ΔgRNA control line #34. Each row within a genotype corresponds to an independent biological replicate.

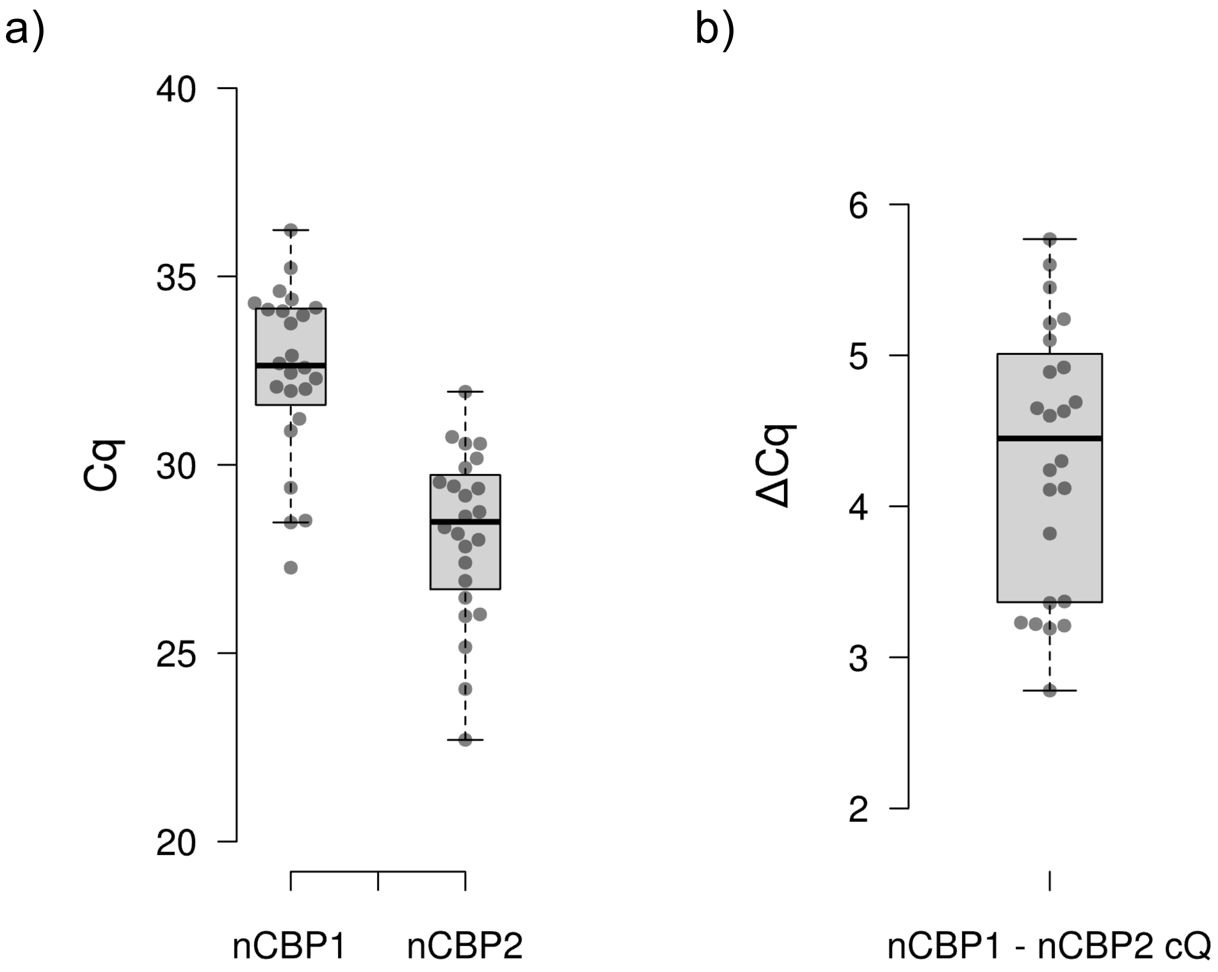

Figure S4.

a) Observed cycle quantitation (Cq) values for *nCBP1* and *nCBP2* across from across five qPCR experiments examining tissue culture leaf, greenhouse leaf, and CBSV infected storage root material. Threshold values for Cq selection were chosen based on those automatically selected by qPCR software for *nCBP-2* and then applied to *nCBP-1*.
b) Differences in *nCBP-1* and *nCBP-2* Cq from each tissue sample used in (a).
